# Structural Heart Valve Disease in the Era of Change and Innovation: The Crosstalk between Medical Sciences and Engineering

**DOI:** 10.3390/bioengineering9060230

**Published:** 2022-05-24

**Authors:** Francesco Nappi, Antonio Nenna, Massimo Chello

**Affiliations:** 1 Cardiac Surgery, Centre Cardiologique du Nord de Saint-Denis, 93200 Paris, France; 2 Cardiovascular Surgery, Università Campus Bio-Medico di Roma, 00128 Rome, Italy; a.nenna@unicampus.it (A.N.); m.chello@unicampus.it (M.C.)

## 1. Introduction

In recent years, both cardiology and cardiovascular surgery have witnessed an era of consistently evolving changes which have dramatically transformed the course and management of cardiovascular disease [1,2,3,4,5,6,7,8,9,10,11,12,13,14]. The innovations initially involved the management of coronary disease, followed by aortic valve stenosis and mitral valve disease, with the progressive evolution and support of percutaneous procedures and transcatheter valve therapy [15,16,17,18,19,20,21,22]. In this context, bioengineering has followed a similar evolutionary pathway since its inception as being a science applicable to pathological processes that progressively adapts to new evidence [23,24,25,26,27,28,29]. The grounds of knowledge shared by medicine and bioengineering are providing newer and higher-performing devices; this positive trajectory should be clear to current practitioners and trainees to foster the scientific debate. We are confident that bioengineering will have a role as an ideal partner in the improvement of new transcatheter platforms for structural heart diseases, even in cardiac surgery, besides the widely established advances in the cardiological scenario. Similarly, a crucial role will be played by the use of artificial intelligence in diagnostic imaging and procedural planning [30,31,32,33,34,35,36].

Therefore, computational biomodeling of the heart structure using finite element analysis (FEA) studies can be considered a pivotal method of predicting the complications often associated with the use of new devices, thereby allowing safer procedures for the future. Studies that address the concerns about bulky calcifications characterizing valve stenosis or pathoanatomic phenomena of mitral annular disjunction should be a priority for bioengineering, so that the ongoing paradigm shift which supports the new procedures using transcatheter approaches can be definitively affirmed and supported by scientific planning. We hope that the recent acceleration and the injection of current intellectual resources [24,25,26,27,28,29,30,31,32,33,34,35,36] might be sustained over the coming years to enhance device quality and provide tailored options for patients suffering from structural heart disease.

## 2. Current Scenario and Future Openings

The use of computational biomodeling with FEA in the field of cardiovascular science has emerged as an essential method to obtain valuable data about complicated real-world structures that otherwise would be impracticable to squarely determine [24,25,26,27,28,29,30,31,32,33,34,35,36]. Since the 1980s, this new scenario has mainly been driven by the emergence of percutaneous coronary intervention with novel options for the treatment of coronary heart disease. The new endovascular platforms have evolved rapidly and established themselves as vital cogs in the armamentarium, available to address structural heart disease [37]. In the last 15 years, the innovation has primarily been invested in the management of aortic valve stenosis and subsequently of mitral valve disease with the progressive affirmation of transcatheter valve therapy (TVT) [15,16,17,18,19,20,21,22]. From the first experimental study by Bonhoeffer, who pioneered the transcatheter pulmonary valve implant [2], the use of TVT to treat aortic valve stenosis progressed rapidly. In 2010, the first PARTNER study (Placement of Aortic Transcatheter Valve Trial) reported a series of high-risk patients who were treated using this novel technique as opposed to conventional aortic valve stenosis surgery [15,16,17,18,38,39]. In less than 10 years, the PARTNER II and III studies affirmed the safety and efficacy of transcatheter aortic valve replacement in low-risk patients [40,41,42,43,44,45]. It is conceivable that future generations of transcatheter valves with the advancement of device technology will herald improvements in the hemodynamic profile, longevity, and durability alongside reduced adverse events, even more than what they have technologically achieved in recent years.

In contrast, computational biomodeling using FEA investigation has not followed a similar evolutionary course since its inception as a method of investigating cardiovascular disease, lacking a true affirmation underpinned by gradual and steady progression while revisiting previous evidence that emerged in the early 1990s [23,24,25,26,27,28,29,30,31,32,33,34,35,36]. For this reason, and given the progress made in the field of coronary heart disease and in the treatment of structural heart disease, a dynamic transformation in the trajectory of the well-planned use of bioengineering and its investigation methods would be desirable to identify a new field of interest, which provides an additional stimulus for cardiologists, heart surgeons, and trainees. The proposed challenge is decisive, and the computational biomodeling experts who will accept it will be among the main players in the process of implementing advances applied to cardiology and cardiovascular surgery and will be able to set the “right” course for this change. To make this happen, a “paradigm shift” is required.

## 3. The Paradigm Shift in Cardiovascular Disease

Thomas Kuhn, an American physicist and philosopher, introduced the term “paradigm shift” for the first time in *The Structure of Scientific Revolutions* in 1962 [46]. In this report, the author explained how a process can bring about a transition of the global view, which has been previously and widely accepted, to a new model because of new emerging evidence. The progress of humanity is made possible due to these paradigm shifts that have affected various disciplines. Some of these can be considered as innovative pillars in the evolution of science and the world. Examples include the emergence of Einstein’s theory of relativity and the expansion of the universe in the field of physics, or Keynes’s theories for economics. Certainly, every transition period does not have a rapid evolution, nor is it devoid of tensions that may be linked to a justified uncertainty about the future. It is still true, however, that paradigm shifts illuminate progress and remain a driving force for future evolution.

Cardiology and cardiovascular surgery are not unresponsive to paradigm shifts because these disciplines are constantly open to timely transitions, gradually favored by the innovative spirit of pioneers. Historically, numerous paradigm shifts have revolutionized clinical practice: coronary bypass grafting, heart transplantation, percutaneous coronary intervention, mechanical and bioprosthetic valves, generations of life-saving drugs for heart failure, and mechanical circulatory support [47,48]. The current zenith of these advancements is the emergence of devices used for the treatment of heart valves with TVT.

However, previous advancements were primarily related to surgical technique or bioengineering details, which have made possible all the clinical improvements we have witnessed in recent years. Current clinical problems, such as structural valve degeneration, complications from percutaneous procedures, and long-term effects of bioprostheses, allografts, or xenografts, require a more advanced approach, and in this setting, advanced bioengineering techniques should be used to implement and support clinical advances.

## 4. What Is Next?

We are confident to be able to map a similar pathway for the advent of computational biomodeling using FEA working on predictive models that can offer the potential to reach a turning point for an indisputable affirmation of the new armamentarium for TVT procedures. We are convinced that the time has come to affirm the central role of bioengineering applied to medical sciences. Therefore, with regard to the use of methods based on the geometric algorithmic prediction of the stress and deformation coefficients in intricate structures, cardiologists, cardiac surgeons, and trainees must perceive the paradigm shift as an advantage, to be faced without prejudice, and consider it a fundamental step in the advancement of cardiological science and as a relevant opportunity [20,23,24,25,26,27,28,29,30,31,32,33,34,35,36]. In fact, thus far, FEA has been perceived with distrust in the field of cardiology and cardiac surgery, thus exerting little influence on clinical practice. This attitude is due, at least in part, to the fact that surgeons have a suspicion towards speculative data with no related clinical evidence. In this context, the shift towards the new concept of the extensive use of computational biomodeling coupled to the percutaneous TVT approach is probably neither alone nor final. However, every day, we are confronted with a rapid innovation that has always been and will continue to be a valuable addition to this profession [37,46,47,48].

## 5. Bioengineering Application: Mitral Valve Pathophysiology

When the use of FEA, the science of geometric algorithms, first emerged as a revolutionary method for obtaining precious information on complicated real-world systems, it was predestined, in future, to change the concept of prediction of stress and strain coefficients in complex structures, such as the determination of principal stresses of mitral valve leaflets. The landmark development of a three-dimensional finite element model of the mitral valve incorporated all essential anatomic constituents including regional tissue thickness, collagen fiber orientation, and related anisotropic material properties [23]. Working on this computational biomodeling, investigators demonstrated that the combination of annular and papillary muscle contraction led to a beneficial effect on valve function [23]. Substantial evidence has made a significant contribution to understanding the main direction of stress exercised in the systolic–diastolic mechanism of mitral valve functioning. The stress developed in the mitral valve during the cardiac cycle was well correlated with the orientation of the collagen fibers. For the first time, through the use of FEA investigation, investigators revealed that early coaptation of the leaflets was closely related to annular contraction, thus promoting valve closure [23]. Again, the contraction of the papillary muscles worked to increase the stress on the chordae tendinae and on both leaflets, ensuring the separation of the latter during the systole phase. The combination of the two mechanisms, by adding these effects, resulted in a more uniform distribution of stress in the mitral valve [23].

One of the main pillars of the biomechanics of the mitral valve, corroborated by the use of FEA analysis, is based on solid evidence that, during valve closure, the anterior leaflet undergoes large anisotropic strains with a markedly high level of peak stretch rates. The rapid elongation to which the collagen fibers are subjected is followed by a plateau phase that leads to a relatively constant state of deformation of the anterior flap of the mitral valve. The plateau phase thus generated represents the result of the complete straightening of the collagen fibers of the anterior leaflet when the valve is closed. The precise arrangement of the collagen fibers that constitute the supporting structure of the mitral valve suggests that they are designed to allow the coaptation of the leaflets, which is linked to a dramatic increase in the rigidity of the collagen architecture. This stiffness is necessary to prevent further deformation of the mitral valve leaflets, which would lead to valve regurgitation [49].

We learned that patients who receive mitral valve replacement surgery disclose acceptable results despite the high operative risk [19,21,22]. Evidence suggests that, although mitral valve repair in degenerative disease confers better long-term survival than replacement, recipients of surgical repair constitute a benchmark for transcatheter mitral valve-in-valve therapy [19]. However, the wide use of TVT is limited by long-term durability concerns, thereby denying access to younger patients with an intermediate to low risk. Expansion of the procedure to include these patients involves exposing them to the unpredictability of long-term effectiveness [21,22,50]. In this context, the use of FEA biomodeling allows the development of predictive models, for short- and long-term follow-up by means of computed biomodeling applied to TVT. Application of FEA biomodeling can be clinically validated through a comparative analysis with a dynamic computed tomography scan, magnetic resonance, or three-dimensional transesophageal echocardiographic reconstruction of the mitral valve [23,49,51,52,53,54,55,56,57].

Patients who are managed with the use of TVT therapies are subject to higher levels of strain of valve leaflets and attachment of the stent in the trigonal area during the systole as the mitral valve bulges into the left atrium [53]. Likewise, FEA is able to anticipate these biomechanical disturbances that are developed in quasi-static boundary conditions and during fatigued dynamic stress [54], both potentially responsible for an early risk of structural valve deterioration. This process is likely made more noticeable during crimping movements in dispensing systems, especially with the latest TVT generations with thinner leaflets [21,22].

During the transcatheter edge-to-edge procedure, the mechanical behavior of the mitral valve differs between leaflets and the connective scaffolding. Prot et al. suggested that the current trend of biomodeling mitral valve leaflets involves using anisotropic hyper-elastic materials with a favored anisotropic orchestration, which is defined by a single collagen family within an isotropic matrix [54]. Understanding how edge-to-edge works is critical for lasting results because during this procedure, a scallop of the anterior leaflet is attached to its counterpart. Evidence from in vitro studies of mechanical testing has increased concerns about the active elements present in the mitral valve leaflets. In particular, the attendance of pre-strains contributing to physiological deformations during the peak systole of the mitral valve disclosed deficiency as regards biomodeling in numerical studies of the mitral apparatus. Of note, two independent studies [49,55] performed on porcine anterior mitral leaflets using a simulator reported measures of circumferential and radial strains ranging between 15% and 40% at the peak systole. Krishnamurthy et al. [56] calculated these strains using a linear inverse finite element technique aimed to assess the material stiffness of bovine anterior leaflets and suggested that the underestimation of leaflet stiffness was to be considered possible. Transesophageal echocardiographic biomodeling using FEA can measure mitral stress/strain and may predict clinical evolution. Simultaneous predictive biomodeling using FEA associated with computerized three-dimensional imaging can be helpful in forecasting the favorable achievement of long-term TVT implantation [30].

## 6. Future Perspectives

The interdisciplinary heart team represents an opportunity to discuss and reflect on the changes being implemented. Bioengineers should receive the opportunity to foster the debate about how to optimize the heart computational biomodeling paradigm shift program or what additional initiative may be needed to keep abreast with the latest trends. Finally, residents and fellows should make the most of the opportunity for change during their training period with continuing education, including innovation-focused conferences and seminars.

## 7. Conclusions

The use of computational biomodeling using FEA study has been in a phase of stagnation for too long, so a paradigm shift, if embraced, can offer a beneficial change. Indeed, there is ongoing inertia and an apparent lack of current resources during this static phase. The optimization of transcatheter interventions for structural heart diseases can also be supported by the use of FEA models. It is therefore possible to establish a reliable thinking mold for TVT therapies, to bring significant advantages in the evolution of cardiology whilst promoting collaboration across disciplines. Throughout our careers as professionals, promoting a transition period is useful to nourish our dreams and reaffirm the foundation that medicine is a commitment to curiosity and lifelong learning. Only in this way can our profession in the field of cardiological science be fulfilling and rewarding [58,59].

## Data Availability

No sensitive data was included in the manuscript.
